# A Multiplex Immunoassay Method for Simultaneous Quantification of Iron, Vitamin A and Inflammation Status Markers

**DOI:** 10.1371/journal.pone.0115164

**Published:** 2014-12-19

**Authors:** Eleanor Brindle, Daniel Stevens, Christopher Crudder, Carol E. Levin, Dean Garrett, Chris Lyman, David S. Boyle

**Affiliations:** 1 Center for Studies in Demography and Ecology, University of Washington, 218D Raitt Hall, Box 353412, Seattle, Washington, United States of America; 2 PATH, 2201 Westlake Avenue, Suite 200, Seattle, Washington, United States of America; 3 Disease Control Priorities Network, University of Washington, 325 Ninth Avenue, Box 359931, Seattle, Washington, United States of America; 4 ICF International, 530 Gaither Road, Suite 500, Rockville, Maryland 20850, United States of America; 5 Quansys Biosciences, 365 N. 600 W, Logan, Utah 84321, United States of America; CSIR-INSTITUTE OF GENOMICS AND INTEGRATIVE BIOLOGY, India

## Abstract

Deficiencies of vitamin A and iron affect a significant portion of the world's population, and efforts to characterize patterns of these deficiencies are hampered by a lack of measurement tools appropriate for large-scale population-based surveys. Vitamin A and iron are not easily measured directly, so reliable proxy markers for deficiency status have been identified and adopted. Measurement of inflammatory markers is necessary to interpret vitamin A and iron status markers, because circulating levels are altered by inflammation. We developed a multiplex immunoassay method for simultaneous measurement of five markers relevant to assessing inflammation, vitamin A and iron status: α-1-acid glycoprotein, C-reactive protein, retinol binding protein 4, ferritin and soluble transferrin receptor. Serum and plasma specimens were used to optimize the assay protocol. To evaluate assay performance, plasma from 72 volunteers was assayed using the multiplex technique and compared to conventional immunoassay methods for each of the five markers. Results of the new and conventional assay methods were highly correlated (Pearson Correlations of 0.606 to 0.991, p<.0001). Inter-assay imprecision for the multiplex panel varied from 1% to 8%, and all samples fell within the limits of quantification for all assays at a single dilution. Absolute values given by the multiplex and conventional assays differed, indicating a need for further work to devise a new standard curve. This multiplexed micronutrient immunoassay technique has excellent potential as a cost effective tool for use in large-scale deficiency assessment efforts.

## Introduction

Micronutrient (MN) malnutrition is a significant public health problem that affects all segments of society in high, middle and low income countries. In low income countries, deficiencies in MNs such as iron, vitamin A (VA), iodine, zinc, and folic acid are associated with adverse health outcomes especially in groups such as pregnant women and children [1]–[3]. As a consequence, the focus of many public health initiatives is to prevent mild, moderate, and severe MN malnutrition. While many cost-effective interventions exist for the control of MN malnutrition, including both food fortification and supplementation programs, tools that can effectively assess MN deficiencies are needed to accurately establish the magnitude of the problem and identify subpopulations at greatest risk for prioritizing interventions. Improved MN assessment tools will generate high quality national data on deficiency status that contribute to well-designed control programs [4]. They can also contribute to monitoring the impact of the progress of MN programs after implementation.

In this study we focused on the biochemical detection of VA and iron deficiencies, two of the four most common MN deficiencies, with the others including those of iodine and zinc [5]. MN deficiencies are primarily due to poor diet or malnourishment or both [4]. VA is a critical MN, essential for embryonic development, adult growth and development, cellular differentiation, immune function, reproduction and vision [6]. Therefore pregnant women and young children are especially vulnerable to VA deficiency (VAD). VAD is associated with low dietary intake and sub clinical and clinical deficiency cause increased morbidity and mortality [7]. Worldwide, 190 million preschool-age children and 19.1 million pregnant women are estimated to have low serum retinol [8], and it is estimated that 157,000 children under 5 years of age die each year from VAD-related illness [9]. The diagnosis of clinical VAD can be performed by assessing vision-related symptoms, such as night blindness [10], [11]. Alternatively, clinical and subclinical VAD can be identified by biochemical analysis measuring vitamin A in the form of serum retinol via high pressure chromatography [12], by fluorimetry [13] or by assessing the levels of an accepted surrogate biomarker, retinol binding protein 4 (RBP) [14]. Retinol and RBP are under homeostatic control, and thus are not directly reflective of liver VA stores and are poor indicators of VA excess, but both decrease in the case of VAD [15], [16].

Iron deficiency anemia (IDA) leads to reduced physical activity in adults and impaired brain development in children [17]. IDA global prevalence is even greater than prevalence of VAD, with an estimated 2 billion people affected worldwide [18]. Recent estimates are that annually there are 591,000 perinatal deaths and 115,000 maternal deaths linked to IDA worldwide [19]. A variety of laboratory tests can be used to determine the iron status of an individual. Two biomarkers, ferritin and the soluble transferrin receptor (sTfR) are now commonly used to assess iron status in serum or plasma via enzyme-linked immunosorbent assay (ELISA) based methods [20]–[22]. Serum ferritin, the major carrier protein of iron in blood, is a good indicator of iron storage as its concentration declines early in the development of iron deficiency [23]–[25], but increases with inflammation, independent of iron levels [24]. The sTfR level reflects the functional iron component and its levels increases with the onset of IDA [26]. Elevated sTfR in relation to IDA has been confirmed by the evaluation of stainable marrow iron [27], and sTfR has added value in establishing IDA as, unlike ferritin, it is not influenced by inflammation. However, sTfR is only a marker of iron status in the deficient range, and is not an indicator of iron overload [26]. The combined measurement of both ferritin and sTfR allows the data to be further displayed as the ratio of sTfR/ferritin [28], [29], sTfR/log ferritin [30], or log (sTfR/ferritin) [28] to reflect depletion of body iron stores [26]. For the measurement of iron stores, low serum ferritin is noted in the absence of inflammation as the better predictor than sTfR, which reflects tissue iron deficiency rather than body iron stores [26].

Because RBP is a negative acute-phase protein and ferritin is a positive acute phase protein, both their values are altered by subclinical inflammation [31]. C-reactive protein (CRP) and α-acid glycoprotein (AGP) are biomarkers commonly used to adjust for the influence of subclinical inflammation. Recently, a meta-analysis of datasets of ferritin, CRP and AGP values has shown that correction values for ferritin can be calculated based on the levels of CRP and AGP [32], [33].

These biomarkers are typically measured one at a time via a variety of conventional ELISA kits which, while proven to be accurate, are expensive when used in large volumes. Securing adequately trained and equipped labor needed for screening large specimen sets using conventional ELISA also represents a significant challenge in most laboratories. One study described the construction of several ELISAs to assess RBP, ferritin, sTfR, and CRP [34]. However, this was not multiplexed in the conventional sense and instead four separate ELISA plates were prepared and analyzed. In addition, each assay required that specific dilutions were used.

The need for relatively large volumes of plasma or serum can present further logistical and financial complications if venous blood is the sample type used. Venous blood draws may not be accepted by participants or guardians of children, and appropriate transport and processing further increases costs and stability risk to samples. Smaller volumes of specimens collected by less invasive methods, such as finger sticks and collection as dried blood spots, have been shown to be effective for VA analysis [35], but multiple conventional ELISAs may require larger sample volumes than these methods can provide. In addition, some countries now restrict or prohibit the transport of specimens out of the study country, so researchers must perform analysis of specimens in country where shipping and customs costs for ELISA kit importation may further increase the cost of the surveillance. Limited funding means that many health agencies and researchers must now analyze smaller data sets, which may not accurately represent the micronutritional status of the target populations.

Multiplex assay methods, in which a single test procedure yields results for multiple analytes, have potential for reducing the labor, supplies, and sample volumes required for MN screening. However, some existing multiplex platforms are not well-suited to use in low to middle income country laboratory environments. Suspended microscopic bead-based assay platforms [36], like those offered by Luminex (Austin, TX, USA), rely on fluidics systems that require significant routine maintenance and maintenance supplies (e.g. sheath fluid) for proper operation. These systems operate on very different principles from the conventional ELISA protocols with which most lab staff are already familiar, and thus require extensive specialized training and additional equipment. They also involve a longer process of data acquisition, in which bead arrays are passed by a laser in sequential fashion, making even brief interruptions in power supply a significant problem. Planar arrays, in which the assay reactions occur in discrete areas of a rigid, 2-dimensional surface [36], generally require less routine maintenance than bead-based systems and can offer a more familiar assay procedure for those trained in conventional ELISA, particularly when the array is immobilized on the surface of a microtiter plate. One such platform, the Meso Scale Discovery (Rockville, MD, USA), is based on electrochemiluminescent detection, but the instrument required to quantify the assay signal is expensive. The Searchlight multiplexed assay technology offered by Thermo Fisher Scientific (Walthamstow, MA, USA) uses chemiluminescent detection of signals with equipment that is complex and expensive. However, with other appropriately designed assay platforms, chemiluminescent endpoints for multiplexed reactions can also be quantified using comparatively inexpensive and low-maintenance imaging instruments that would be practical for laboratories with limited resources.

In this study we describe the development of a multiplexed MN assessment tool (MMAT) which permits the concurrent measurement of five analytes relevant to vitamin A and iron deficiency surveillance–AGP, CRP, ferritin, RBP and sTfR–from a single 8 µL volume of serum or plasma. The MMAT uses multiplex assay technology and software developed by Quansys Biosciences, Inc. (Logan, UT, USA) in which capture antibody arrays are printed in distinct regions of the wells of a microtiter plate to create a low density planar array. The process for each region of the array then follows the principles of conventional ELISA technology with a chemiluminescent signal generated at each array spot for quantitative measurement. This format requires only conventional laboratory equipment for ELISA assay preparation, and chemiluminescent reactions are captured via an inexpensive high resolution digital camera-based device. The samples are analyzed and scored *in silico* via an automated platform allowing simple and accurate analysis of 40 specimens in duplicate per plate in a single 3 hour test. Multiple plates may be batched to process large numbers of specimens for a variety of analytes in a simplified flow, saving time and money to generate large datasets. A basic cost analysis of the prototype array was USD$12 per sample compared to a combined cost of USD$34 using five conventional commercial ELISA kits as purchased in the USA.

## Materials and Methods

Testing of the MMAT was undertaken in two distinct phases. Phase 1 of testing, led by Program for Appropriate Technology in Health (PATH; Seattle, WA, USA), included contracting Quansys Biosciences to perform initial development of the multiplex assay method and produce assay kits. Preliminary assessments of the MMAT kits and refinements to the protocol were then performed independently by a lab at PATH. Phase 2 of testing included evaluation of MMAT performance as compared to conventional ELISAs, and was conducted by the Biodemography Lab at the University of Washington's Center for Studies in Demography and Ecology (Seattle, WA, USA).

### Phase 1

#### MMAT assay principles and development

Quansys Biosciences were contracted to conduct the initial assay development using their proprietary Q-Plex technology to construct low density protein arrays with capture antibodies printed on the base of a flat bottomed microtiter plate well [37], [38]. The binding of detection moieties, e.g. a biotin labeled antigen or secondary antibody, is measured via the chemiluminescence produced by streptavidin horseradish peroxidase (S-HRP) in the presence of a luminol-based substrate. The intensity of chemiluminescence from each array was measured using the Q-View chemiluminescent imager (Quansys Biosciences). Data were then analyzed using Q-View software (Quansys Biosciences). To assess quantitation and sensitivity of each assay across their respective ranges, the analyte's standard dilution series data was analyzed using a 5 parameter logistic (5-PL) regression.

Phosphate buffered saline (PBS) was purchased from Sigma Aldrich Chemical Company (St Louis, MO, USA). Capture antibodies were purchased from HyTest Ltd. (Turku, Finland) including CRP (4C28 C6), RBP 4RB2 RB42, sTfR (4Tr26 13E4) and Sigma Aldrich for AGP (GW22927F). A pair of capture (F23) and detector (F31) antibodies to ferritin was also purchased from HyTest (4F32). Purified sTfR (8Tr56), CRP (8C72) and RBP (8RF9) antigens were purchased from HyTest, Ferritin (RP-87068) was from Thermo Fisher and AGP (G9885) from Sigma Aldrich. The EZ-Link Sulfo-NHS-LC-Biotin labeling kit was purchased from Thermo Fisher. Standards curves were prepared from the Liquichek Immunology control 3 (Bio-Rad Laboratories, Inc., Hercules, CA) as described below.

**Figure 1:**
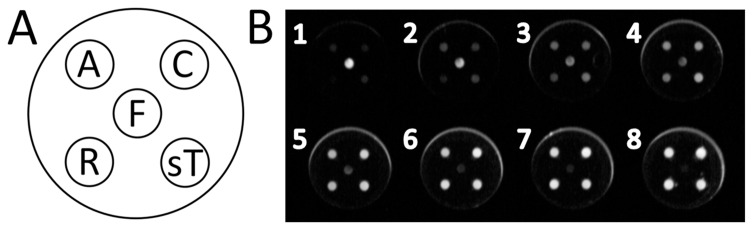
Fig. 1A. The arrangement of multiplexed assays in each well. A, alpha-1-acid glycoprotein; C, C-reactive protein; F, ferritin; R, retinol binding protein 4; sT, soluble transferrin receptor. **Fig. 1B. An image of 8 array wells demonstrating the chemiluminescence generated from assay spots.** The top left hand well (well 1) indicates the highest concentration of standard followed by six subsequent two-fold dilutions (wells 2 through 7). The final lower right hand well (well 8) is the buffer-only negative control.

For this study, mouse monoclonal antibodies for each of the 5 analytes were printed in varying concentrations as discrete regions onto the bottom of flat well microtiter plates (Sigma Aldrich) to assess their performance as capture agents (Fig. 1A). The performance of each capture antibody was initially screened via a simple competitive assay which measured the linear depletion of biotinylated antigens in the presence of increasing amounts of unlabeled antigen (see Fig. 1B). Aliquots of each purified antigen were labeled with biotin (EZ-Link Sulfo-NHS-LC-Biotin kit) according to the user instructions for use in the competitive assay format.

The assay for ferritin in a competitive format was not sensitive enough as its working concentration is approximately ten times less than that of the other analytes used in this study. Therefore a more sensitive sandwich immunoassay [39] was developed for ferritin whereby a secondary ferritin monoclonal antibody (F31) was used to bind to ferritin already captured by the fixed capture antibody (F23). This secondary antibody was also labeled with biotin (EZ-Link Sulfo-NHS-LC-Biotin kit) and therefore could be detected by same the S-HRP reporter method. In this format the ferritin assay had sufficient sensitivity over physiologically relevant concentration ranges. All assays were then integrated together as five spot multiplexed arrays printed in single wells of a 96 well plate (Fig. 1).

Quansys Biosciences supplied the MMAT assays to PATH in a kit format with the five assays multiplexed in each well for evaluation using serum and plasma specimens. The kits contained prepared plates, sample diluent and lyophilized diluent additive containing the biotin labeled antigen competitors (AGP, CRP, RBP, sTfR) and detection mix containing labeled secondary antibody (ferritin), wash solution concentrate, streptavidin-horseradish peroxidase (S-HRP) conjugate and chemiluminescent substrate.

#### Conventional immunoassays

Conventional ELISA kits widely used in previous MN status surveillance efforts [35], [40] were selected as the reference method to which the new multiplex method would be compared (Table 1). A kit from Ramco Laboratories Inc. (Stafford, TX, USA; cat # TFC-94) was used to measure sTfR. According to the manufacturer's evaluation of assay performance, the limit of detection (LOD) is 0.07 µg/mL, intra-assay CV for six serum specimens assayed in 10 replicate wells ranged from 2.4% to 5.8% for mean sTfR concentrations ranging from 5.5 to 14.9 µg/mL, and inter-assay CV, evaluated by assaying 12 serum specimens in duplicate over five weeks, ranged from 3.6%–8.1% for concentrations from 4.1 to 16.1 µg/mL. Ramco Laboratories also supplied the ferritin kit (cat #S-22). The LOD is 0.59 ng/mL. Intra-assay CV, evaluated using 8 assay runs, ranges from 4.7%–9.6% and inter-assay CV, evaluated using 12 assay runs, ranges from 6.8% to 8.7%. RBP4 was measured using the Scimedx Scanlisa RBP EIA (Scimedx Corp., Denville, NJ, USA; cat #RBP196), originally developed and validated at PATH [41]. The assay LOD is 1.1 µg/mL. Intra-assay CV is 6.7% and inter-assay CV is 8.9%. AGP was measured using an ELISA reagent kit supplied by Genway Biotech, Inc. (San Diego, CA, USA; cat# 40-288-22927F). The LOD is 0.00391 µg/mL. Intra- and inter- assay CVs for controls prepared in assay buffer from pure human AGP and run in duplicate were 6.2% and 7.1%, respectively, for the low (0.0087 µg/mL) control and 4.0% and 0.5% for the high (0.0318 µg/mL) control (n = 10 plates). An assay kit supplied by IBL-America (Minneapolis, MN, USA; cat # IB79102) was used to measure CRP. The kit had a LOD of <1 µg/mL. Intra-assay CV (n = 10) ranged from 5.12% to 6.84% and inter-assay CV (n = 7) ranged from 11.6%–12.7%. Each immunoassay was carried out according to the manufacturer's specific instructions. Spectral data was read using a SpectraMax M2 reader (Molecular Devices, Sunnyvale, CA, USA).

**Table 1:** Assay precision and limits of detection (LOD) for conventional ELISAs.

Assay	Supplier	LOD	Control	Intra-assay precision (mean, CV)	Inter-assay precision (mean, CV)
AGP (µg/mL)	Genway	0.0039	High	0.0318, 4.0%	0.0318, 0.5%
			Low	0.0087, 6.2%	0.0087, 7.1%
CRP (µg/mL)	IBL America (PATH lab)	<1	High	48.3, 6.84%	67.2, 12.7%
			Medium	—	31.0, 11.6%
			Low	5.2, 5.12%	4.3, 14.3%
	in-house (UW lab)	0.00015	High	0.0063, 2.7%	0.0063, 9.9%
			Low	0.0033, 3.5%	0.0033, 9.4%
Ferritin (ng/mL)	Ramco Laboratories, Inc.	0.59	High	208.0, 4.7%	219.0, 8.7%
			Medium	54.5, 9.6%	55.7, 7.7%
			Low	10.4, 5.8%	11.0, 6.8%
RBP (µg/mL)	Scimedx Corp.	1.1	†	8.9–37.8, 6.7%	8.9–37.8, 8.9%
sTfR (µg/mL)	Ramco Laboratories, Inc.	0.07	High*	14.9, 3.5%	16.1, 5.0%
			Medium	9.4, 5.8%	7.3, 7.3%
			Low	5.5, 2.4%	4.1, 4.4%

Coefficients of variation (CV) for the conventional ELISA kits against which the multiplexed micronutrient assessment tool (MMAT) was compared. AGP, alpha-1-acid glycoprotein; CRP, C-reactive protein; RBP, retinol binding protein 4; sTfR, soluble transferrin receptor.

† For RBP, precision given as average intra- and inter-assay CVs in the calibrated range.

*For sTfR, 3 values are presented from the 6 reported for intra- and 12 provided for inter-assay CV in the kit insert.

#### MMAT assay protocol optimization and preliminary evaluation

A commercially available control, the Liquichek Immunology control level 3 (Bio-Rad), was used to produce standard curves for the MMAT. Liquichek 3 is a pooled serum-based matrix and has previously been described to contain the biomarkers within the critical ranges required to assess VAD, IDA and inflammation [34]. In the prototype design work the median values supplied with the product insert were used for AGP, CRP and ferritin and the concentrations of RBP and sTfR determined via conventional ELISA kit measurement. However, because the Liquichek controls are derived from pooled human serum, there is lot-to-lot variance and values specified in the product insert vary by analysis method used. Before analyzing samples with both the conventional ELISA kits and MMAT, the concentration of each of the five analytes in a new lot of Liquichek 3 was estimated using the conventional ELISA kits. Liquichek 3 samples were prepared in either two or three dilution sets that reflected the mid-range concentration of the standard curve for each assay. Each dilution of Liquichek 3 was screened ten times per plate, and the average resulting concentrations were used as known doses to form the basis for MMAT calibration.

Using the Liquichek 3 dilution series, the general performance of the MMAT for low to high concentrations of the pooled analytes was assessed. A series of serial dilutions of the Liquichek 3 were used to establish the LOD to ensure that the assays could accurately detect low concentrations of each analyte below the thresholds used to determine sufficiency/deficiency. The data sets used to estimate the analyte concentrations for the Bio-Rad Liquichek 3 standard curve data were also used to assess inter- and intra-assay coefficient of variation (CV). The high, medium and low concentrations of the analytes were used for both measurements. The inter-assay CV used 3 dilutions sets of the Liquichek standards. For the intra-assay CV individual values were pooled from 3 plates (9 single data points per analyte concentration). Assay linearity was assessed by assaying three serum samples at 1∶2, 1∶4, 1∶8 and 1∶16 and comparing results at the 1∶2 dilution series to results at each subsequent dilution.

#### Serum and plasma specimens

For Phase 1 of testing, we purchased specimen sets from eight adults as serum, lithium heparin plasma and K-EDTA plasma from Bioreclamation Inc. (Westbury, NY, USA). Each sample was centrifuged at 1,500 rpm (approximately 670×*g*) in a swing-bucket centrifuge for 10 minutes at 4°C and multiple 450 µL aliquots were stored at −80°C until use. Preliminary testing of MMAT performance with a variety of sample types was conducted by comparing MMAT results with conventional ELISA kit measurements from these 8 matched specimens. The MMAT protocol optimization process included trouble-shooting sample matrix-dependent differences in concentration (described in detail below) by adding varying doses of EDTA similar to the concentrations expected in samples prepared from K-EDTA plasma tubes to the diluted serum and plasma specimens. Effects of added EDTA were evaluated by comparing MMAT and monoplex results from EDTA spiked and un-spiked specimens.

#### MMAT assay procedure

Standard curves for each analyte were prepared by diluting the Liquichek 3 by 1 in 10 in sample diluent and then as a further series of six twofold dilutions to create a seven point dilution set in addition to a sample diluent only negative control (see Table 2). 50 µL of serum or plasma specimens diluted 1∶20 in sample diluent were added to the plate in duplicate wells.

**Table 2:** Standard concentrations.

	AGP µg/mL	CRP µg/mL	Ferritin ng/mL	RBP µg/mL	sTfR µg/mL
Neat	745±92 (941)*	48.4±3.9 (44.8)*	694±106 (314)*	61.6±8.4 (N/A)*	12.4±1.4 (N/A)*
Standard 1	74.50	4.84	69.4	6.16	1.24
Standard 2	37.25	2.42	34.7	3.08	0.62
Standard 3	18.63	1.21	17.35	1.54	0.31
Standard 4	9.31	0.605	8.68	0.77	0.16
Standard 5	4.66	0.303	4.34	0.385	0.08
Standard 6	2.33	0.151	2.17	0.193	0.04
Standard 7	1.16	0.076	1.08	0.096	0.02
Standard 8	0	0	0	0	0

The revised concentration of the Liquichek 3 control analytes in the test well as measured via the conventional ELISAs (mean ± SD). The standard spans points 1–7 of subsequent twofold dilutions with 8, a diluent only negative control, completing the set. AGP, alpha-1-acid glycoprotein; CRP, C-reactive protein; RBP, retinol binding protein 4; sTfR, soluble transferrin receptor.

* The mean concentrations provided with the Liquichek 3 product insert.

After addition of the standards and samples, each plate was incubated at room temperature on a flatbed shaker (Thermo Fisher) at 500 rpm for 1 hour. The wells were then aspirated and washed 5 times with 300 µL of 1x PBS (ELx50 plate washer, BioTek Instruments, Inc., Winooski, VT, USA). A 50 µL aliquot of the detection mix was then added to each well and the plate then mixed for a further hour and then washed as described above. Labeling was performed by adding 50 µL S-HRP to each well and shaking for 15 minutes at 500 rpm. The substrate parts A and B were mixed in equal volumes and 50 µL of the mixture was added to each well. Each plate was then imaged three times using the Q-View imager with exposure times of 60, 150 and 300 seconds respectively. Using Q-View software the images were then automatically overlaid and the chemiluminescent intensity of each spot, in each well measured. The software was programmed to create standard curves using a 5-PL regression for the 5 biomarker standards based on the concentrations derived from monoplex analysis (Table 2). The concentration of each analyte in each test well was then automatically generated using the standard curves as reference.

### Phase 2

In phase 2 of testing, a set of 72 specimens (lithium heparin plasma) from 36 males and 36 females, procured from Bioreclamation, was used to evaluate MMAT performance using conventional ELISAs as a gold-standard. Samples were prepared and stored as described above. These 72 lithium heparin plasma specimens were assayed in the Biodemography Lab at the University of Washington using the MMAT assay procedure as described above, but with the addition of 10 mM EDTA to the sample diluent, as was indicated by the results of Phase 1 testing, described in more detail below.

The same monoplex assays described above for Phase 1 were used for sTfR, RBP and AGP. Bioreclamation provided conventional assay data for ferritin. To reduce costs, the CRP ELISA kit used in Phase 1 was replaced with an in-house sandwich ELISA using monoclonal capture (clone C5) and detection (clone C6) antibodies (Meridian Life Science, Inc., Cincinnati, OH, USA) [42]. The CRP assay has high sensitivity, with a limit of quantification of 0.00015 mg/L. For plasma controls, intra- and inter-assay CVs were 3.5% and 9.4% respectively for the low (0.0033 mg/L) control, and 2.7% and 9.9% for the high (0.0063 mg/L) control (n = 11 plates). Reactions for all monoplex assays conducted in the University of Washington lab were quantified using a BioTek Synergy HT microplate reader (BioTek Instruments).

Results from the conventional ELISAs and the MMAT for the set of 72 heparinized plasma specimens were compared by a variety of methods. Comparisons by Pearson correlation and scatter plots were used to evaluate whether results from the assays covaried. Absolute levels were compared by Bland–Altman analysis [43], [44] and by expressing results from the MMAT assay as a percentage of the results from conventional assays.

## Results

Final concentration values as determined by conventional ELISAs for each analyte in the Liquichek 3 control are listed in the top row of Table 2. The average concentration of AGP in the Liquichek 3 control was described by the manufacturer as being 941 µg/mL. The concentration of AGP Liquichek 3 control using the GenWay assay was established by making three dilutions of 1∶5000, 1∶25000 and 1∶125000. In this analysis the mean ±SD concentration of AGP was estimated to be 745±92 µg/mL. The average CRP concentration for Liquichek 3 control as reported by the manufacturer was 44.8 µg/mL. The dilution series for use with the CRP assay were 1∶1, 1∶2 and 1∶4 and the final concentration established to be 48.4±3.9 µg/mL. The mean concentration of ferritin was 314 ng/mL. Our reanalysis of this gave a markedly higher value of 694±106 ng/mL. The RBP concentration in the Liquichek 3 control was not supplied by the manufacturer's datasheet and in this case a dilution series of 1∶2, 1∶4 and 1∶8 were used based on previous observations (data not shown). The RBP concentration was established to be 61.6±8.4 µg/mL (2.93±0.4 µmol/L). Likewise concentration was not supplied for sTfR. The control was diluted 1∶1 and 1∶2 prior to further dilution according to kit protocol. The sTfR concentration was estimated to be 12.4±1.4 µg/mL.

**Figure 2:**
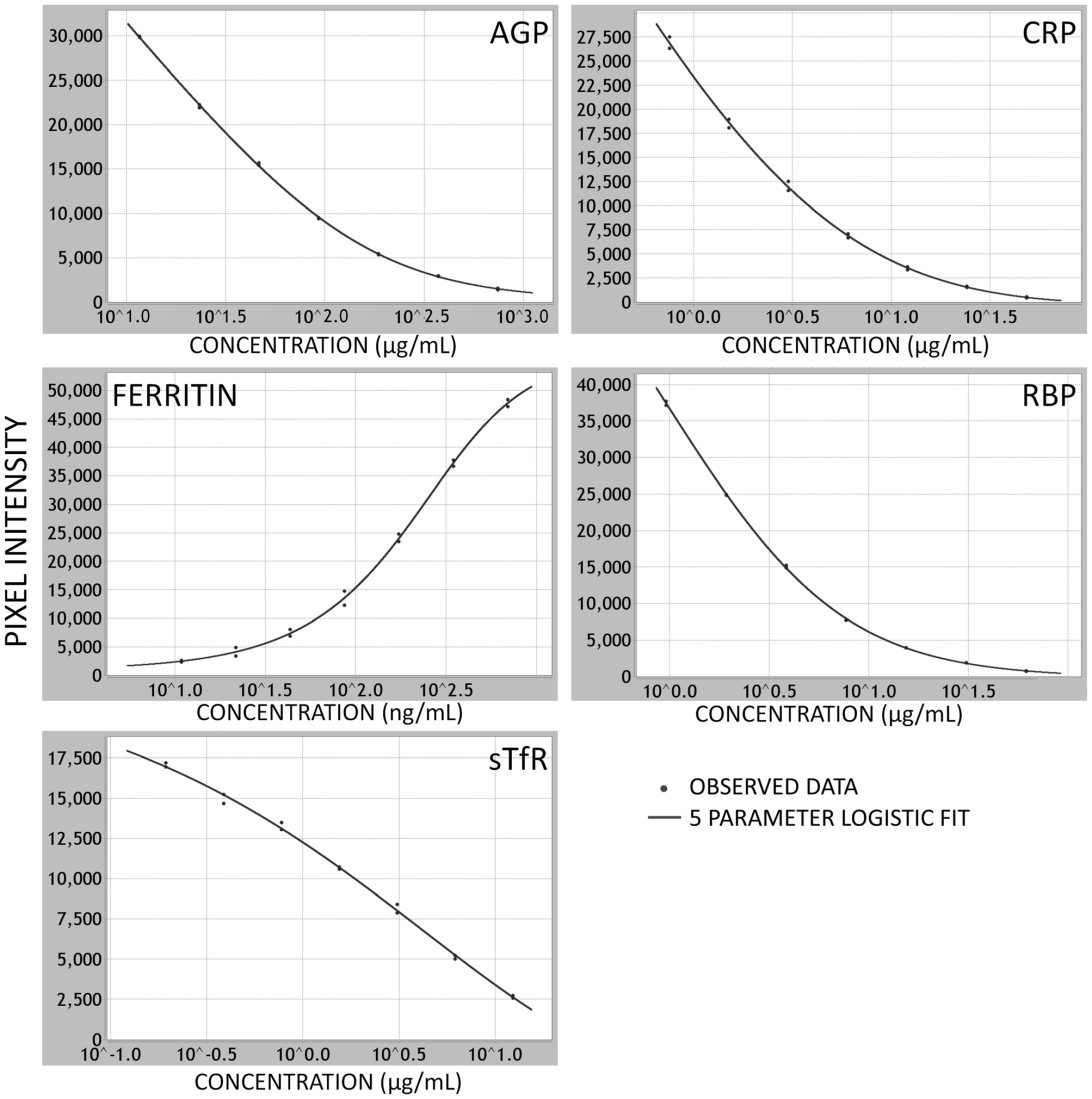
Standard curves for each of the 5 analytes. The standard curves generated after 5-PL regression analysis across seven twofold dilutions of the Liquichek 3 standards for each assay in the multiplexed array. AGP, CRP, RBP and sTfR assay plots are from the basic antigen competitor assays and show concomitant decrease in chemiluminescence as the concentration of analyte is increased reflecting the non-labeled antigen out-competing the biotinylated antigen. In the ferritin sandwich assay, the chemiluminescence increases with analyte concentration reflecting the increased binding of the labeled secondary antibody to the captured ferritin. AGP, alpha-1-acid glycoprotein; CRP, C-reactive protein; RBP, retinol binding protein 4; sTfR, soluble transferrin receptor.

Initial MMAT assay development conducted by Quansys Biosciences resulted in assay kits with a dose-response relationship across the relevant physiological ranges for all five analytes. Using the Liquichek 3 as a standard, the data from the dilution series for each analyte indicated that the performance of each assay in the prototype multiplexed array is sufficient across the relevant concentration ranges required to assess deficiency or inflammation (Fig. 2). Screening of the multiplexed arrays with purified individual antigens did not show any cross reactivity with the other capture assays on the array.

Estimates of LOD and intra- and inter-assay imprecision are shown in Table 3. CVs fell within acceptable ranges (<15%) for all analytes. Tests of assay linearity yielded results ranging from 80% to 117% with no evidence of systematic non-parallelism across dilutions for any analyte (Table 3).

**Table 3:** Assay precision, ranges, lower limits of detection (LOD) and assay linearity for MMAT.

Analyte and Cutoff Value	LOD	Control	Intra-assay precision (mean, CV)	Inter-assay precision (mean, CV)	Assay Linearity	
AGP (µg/mL) (>1000 µg/mL indicates inflammation) [33]	2.98	High	450.7, 3.0%	460.9, 7.9%	1∶2	87%
		Medium	114.1, 0.6%	113.7, 11.2%	1∶4	99%
		Low	27.3, 6.5%	29.2, 13.9%	1∶8	98%
					1∶16	101%
CRP (µg/mL) (>5 µg/mL indicates inflammation) [33]	0.41	High	52.3, 4.9%	50.3, 8.8%	1∶2	101%
		Medium	12.1, 0.6%	12.2, 5.6%	1∶4	100%
		Low	3.4, 4.2%	3.4, 9.3%	1∶8	92%
					1∶16	89%
Ferritin (ng/mL) ID cutoff <15 ng/mL (under 5 years, <12 ng/mL) [33]	1.5	High	279.4, 4.1%	279.4, 9.3%	1∶2	117%
		Medium	70.1, 6.1%	70.1, 12.8%	1∶4	85%
		Low	31.5, 14.8%	31.5, 14.1%	1∶8	111%
					1∶16	114%
RBP4 (µg/mL) VAD cutoff <14.7 µg/mL (0.7 µmol/L) [41]	0.89	High	41.3, 11.3%	42.4, 9.3%	1∶2	96%
		Medium	10.7, 0.7%	10.8, 8.7%	1∶4	109%
		Low	3.2, 2.2%	3.0, 5.3%	1∶8	107%
					1∶16	97%
sTfR (µg/mL) ID cutoff >8.3 µg/mL [34]	0.08	High	3.6, 2.0%	3.4, 7.3%	1∶2	110%
		Medium	0.9, 0.3%	0.9, 9.3%	1∶4	98%
		Low	0.2, 11.1%	0.2, 13.5%	1∶8	91%
					1∶16	80%

AGP, alpha-1-acid glycoprotein; CRP, C-reactive protein; RBP, retinol binding protein 4; sTfR, soluble transferrin receptor; ID, iron deficiency; VAD, vitamin A deficiency; MMAT, multiplexed micronutrient assessment tool; CV, coefficient of variation.

**Figure 3:**
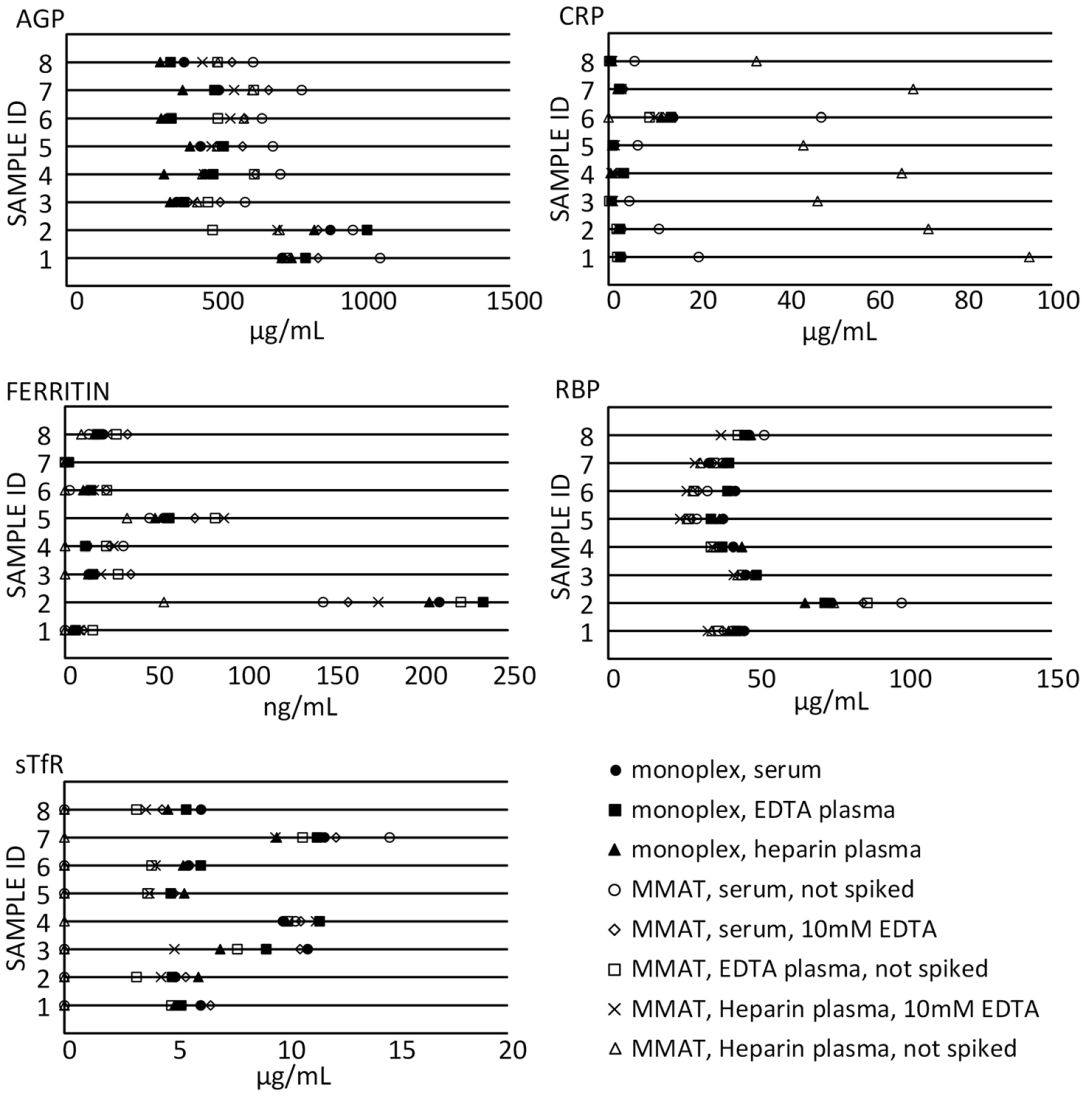
Effects of sample type and addition of EDTA to diluent. AGP, alpha-1-acid glycoprotein; CRP, C-reactive protein; RBP, retinol binding protein 4; sTfR, soluble transferrin receptor.

Phase 1 MMAT testing conducted using a set of 8 matched serum, heparin plasma and EDTA plasma revealed significant differences in concentration by sample matrix for some sample type-analyte combinations (Fig. 3). In particular, MMAT CRP, ferritin and sTfR concentrations from serum and heparin plasma specimens differed more from monoplex results than did MMAT results for EDTA plasma, and in some cases were outside the assay range at the same dilution that provided valid results for EDTA plasma specimens. When the dilutions of serum and heparin plasma were prepared in sample diluent containing 10 mM EDTA, a concentration similar to that of EDTA plasma samples, results were in closer agreement with monoplex assays and results for EDTA plasma in the MMAT. The presence or absence of 10 mM EDTA in the sample diluent did not show marked effects on AGP or RBP results.

After optimizing the MMAT protocol for use across sample types, results from this small set of specimens indicated that a dilution of 1∶20 was effective for these samples for all analytes. 160 µL of 1∶20 pre-diluted samples were prepared to provide sufficient volume to add 50 µL per well in duplicate using a multi-channel pipette. Thus, the total initial sample volume required to measure all five analytes was only 8 µL.

In the following evaluation, 72 heparin plasma specimens were measured in the MMAT assay and conventional monoplex assays. At a dilution of 1∶20, the MMAT produced results within range for all specimens and all analytes, with the exception of 9 specimens for which ferritin results were below the LOD. Those 9 specimens were assayed again at a 1∶10 dilution, and were quantifiable at that dilution, but still fell near the lower end of the Quansys ferritin assay range.

**Figure 4:**
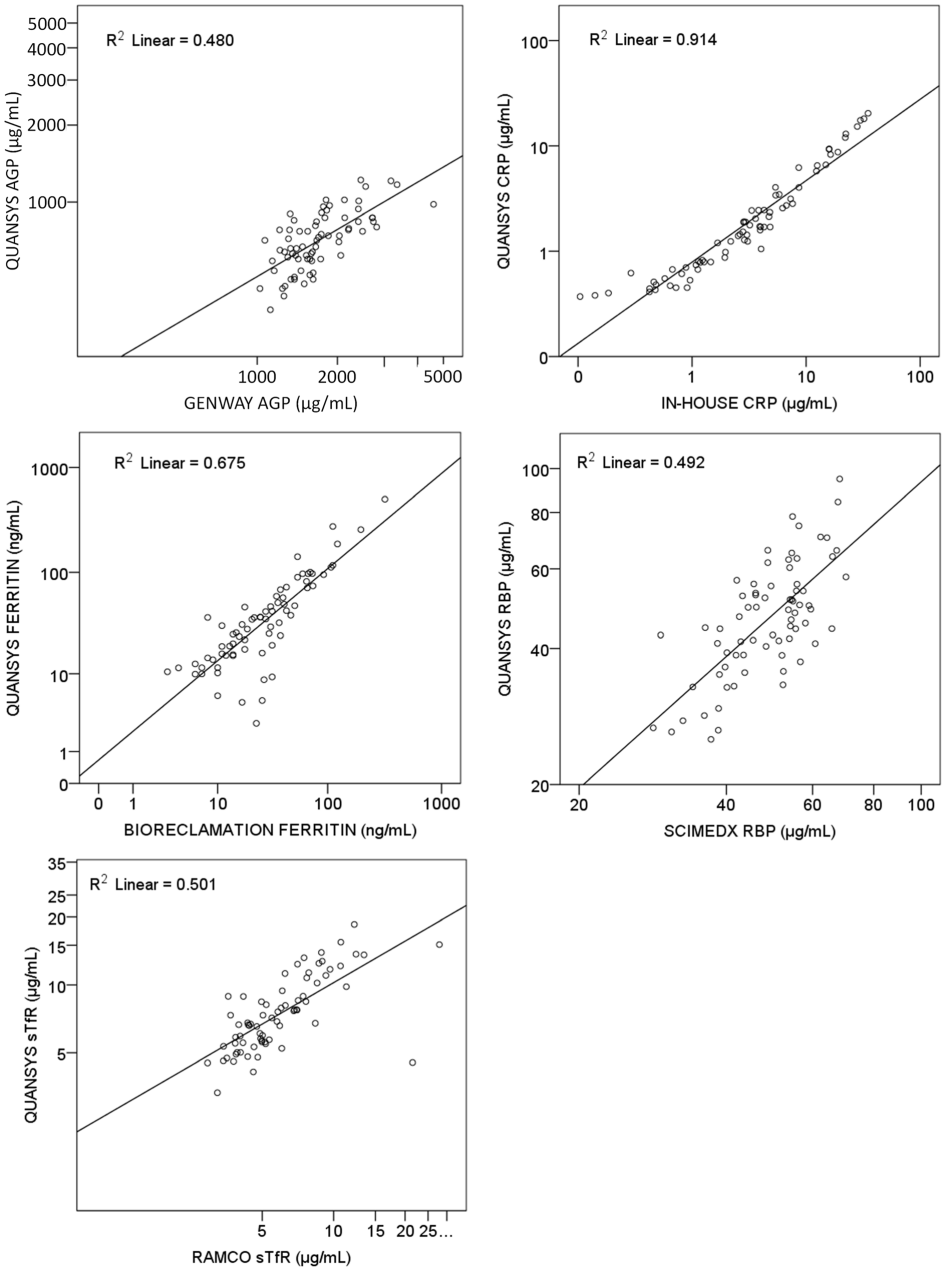
Scatterplots of Quansys MMAT results versus conventional assay results. Concentrations of each analyte as measured in the Quansys MMAT (y-axes) plotted against concentrations measured using conventional assays (x-axes) for 72 plasma specimens (log scales). Solid line is linear regression. AGP, alpha-1-acid glycoprotein; CRP, C-reactive protein; RBP, retinol binding protein 4; sTfR, soluble transferrin receptor.

**Figure 5:**
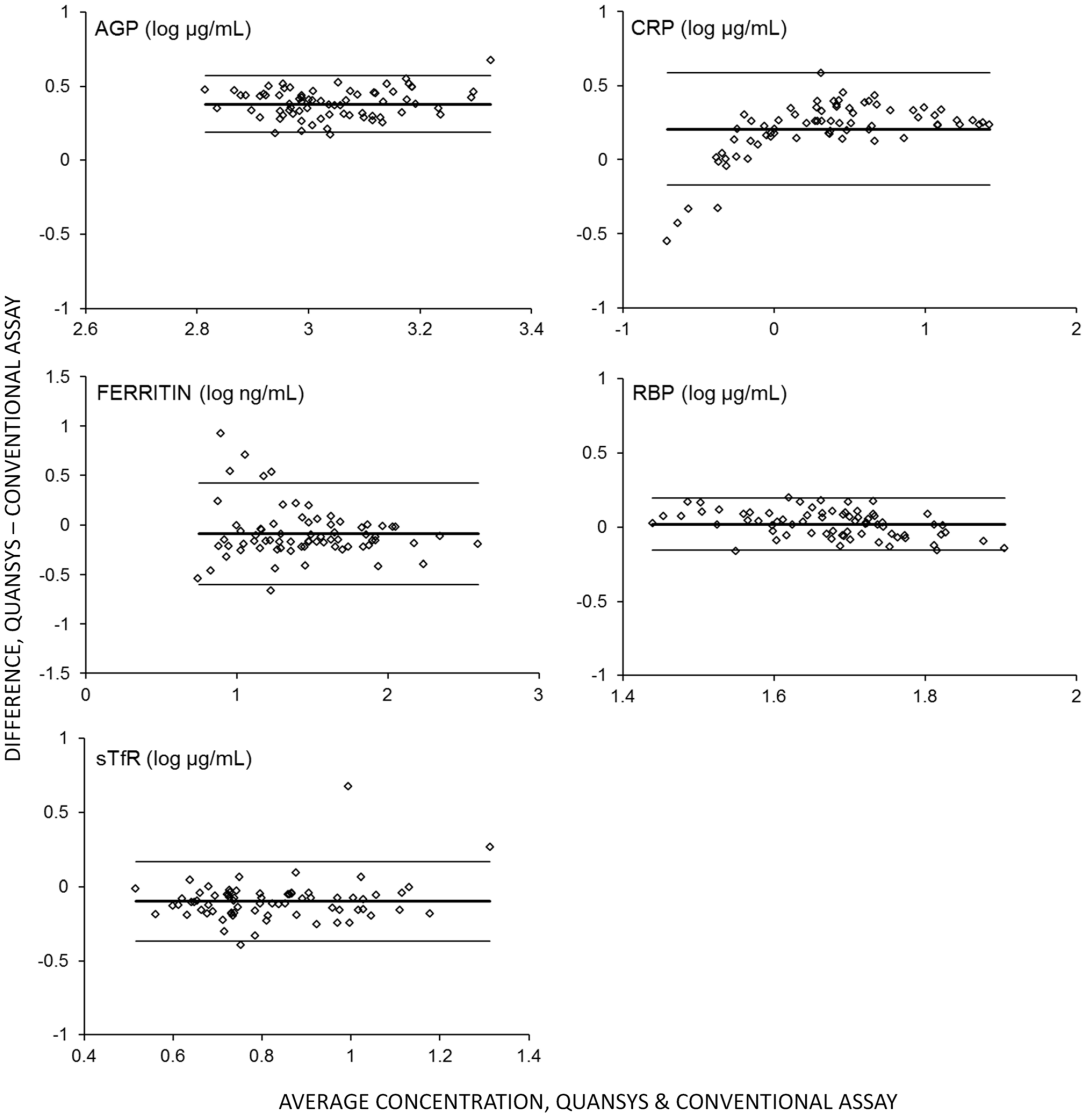
Bland-Altman analysis. Bland-Altman plots of differences between log concentrations (y-axes) versus the average concentrations (x-axes) for each analyte in 72 plasma specimens assayed using the Quansys MMAT and conventional assays. Center solid line indicates mean difference; upper and lower solid lines indicate the 95% confidence interval. AGP, alpha-1-acid glycoprotein; CRP, C-reactive protein; RBP, retinol binding protein 4; sTfR, soluble transferrin receptor.

Results of the new MMAT assay and conventional methods were highly correlated (Table 4, Fig. 4). Pearson Correlations ranged from 0.606 to 0.991 and all correlations were significant (p<0.0001). Concentration estimates from the MMAT expressed as a percent of results from monoplex assays showed differences in absolute value for some analytes (Table 4), with the largest difference found in the AGP results. Bland-Altman analysis (Fig. 5) did not show evidence of bias in the relationship between absolute values across concentration, suggesting that recalibration of the MMAT assay could correct for these differences.

**Table 4:** Comparisons of MMAT and conventional monoplex assay results from 72 heparin plasma specimens.

Analyte	Monoplex assay	Pearson Correlation MMAT & Monoplex	MMAT/Monoplex (%) ± SD^1^
AGP	GenWay	0.674 (p<.001)	42%±12%
CRP	In-house ELISA	0.991 (p<.001)	69%±56%
Ferritin	data provided by Bioreclamation	0.951 (p<.001)	140%±75%
RBP	Scimedx	0.678 (p<.001)	95%±25%
sTfR	Ramco	0.606 (p<.001)	130%±39%

MMAT, multiplexed micronutrient assessment tool; AGP, alpha-1-acid glycoprotein; CRP, C-reactive protein; RBP, retinol binding protein 4; sTfR, soluble transferrin receptor.

1 MMAT/Monoplex (%) ± SD refers to concentration of analyte as measured by MMAT as a percent of that measured in the conventional monoplex assay.

## Discussion

Our evaluation found that this multiplex assay method performed well for simultaneous assessment of 5 analytes, AGP, CRP, ferritin, sTfR and RBP, using only 8 µL of specimen. This method offers a promising alternative to carrying out five individual assays to measure these markers. At approximately USD$12 per specimen, this assay kit costs significantly less than measuring the same five analytes with separate kits, which would total about USD $35 for the commercial conventional assay kits we used here. The MMAT method also takes less time to carry out and uses significantly less sample volume. If a technician were to run five separate conventional ELISAs on a single sample set, a significant amount of time must be taken in preparing samples at the appropriate dilution for each kit, whereas with Quansys assay only one sample dilution preparation step is required. Furthermore, preparation of the standard curve need only be performed once on the Quansys platform as opposed to five separate standard serial dilutions for individual kits. The complexity of preparing multiple dilution series can also create a greater opportunity for catastrophic error in setting up the varied conventional ELISAs. Finally, the Quansys Q-plex platform offers significant reduction of consumables waste, such as pipette tips and wash solution, since only one plate's worth of consumables is used. Overall, the labor, plate and consumable cost savings of the Quansys platform offer a significant advantage over conventional ELISAs.

One limitation of this study, and of the MMAT in its present form, is the assay's calibration. A commercially available control, the Bio-Rad Liquichek Immunology control level 3, was assayed in conventional ELISAs for the five analytes to estimate concentrations that were then used to calibrate the MMAT assays. This method has been used previously [34], but because the relative quantities of each analyte are fixed, this approach does not allow for optimal adjustment of the standard curve ranges to precisely span the relevant clinical range for each of the 5 analyte assays in the panel. Further investigation is required to optimize the assay calibrators using purified or recombinant antigen cocktails. This would provide flexibility to adjust the standard curve to encompass the relevant ranges and therefore minimize the need to retest specimens out of range. This is particularly important for the ferritin assay, for which some samples in our study had to be assayed again at 1∶10 to achieve an accurate measurement. A threshold of 12 ng/mL ferritin for children under 5 and 15 ng/mL for adults is indicative of depleted iron stores [33]. The current LOD for the MMAT ferritin assay (1.5 ng/mL) above those cut-off values when samples are assayed at a 1∶20 dilution. While the assay LOD is adequate to allow quantification of ferritin values at or below the threshold, it would require re-assay of the specimens at a lower dilution. For the four other analytes, the MMAT assay LOD is significantly lower than the relevant threshold values even when the samples are diluted to 1∶20 (Table 3).

Improvements to the calibration system will also require adjustments to produce absolute values that are in agreement with established methods. Differences in absolute value between the conventional ELISAs and the MMAT were consistent across concentration, with very few specimens falling outside the 95% confidence intervals in our Bland-Altman analysis (Fig. 5), suggesting that a simple correction may be successful in producing agreement between methods.

A limitation of our evaluation of this assay method is that the specimens used were not collected from a low or middle income country population where VAD and IDA are known to be prevalent. More work is required to evaluate this assay panel with a larger set of specimens known to represent the full physiological range of the biomarkers measured, and to compare the results to other commonly used methods, such as the assays described by Erhardt *et al*. [34]. However, MN deficiency is not confined to developing world populations, as the 72 specimens used in this work to evaluate the MMAT assay performance did include a broad physiological range, including specimens that would be categorized as indicating deficiencies in both iron and vitamin A, and with specimens in both the high and low ranges for AGP and CRP.

A variety of multiplexed protein array technologies have been introduced over the past several years, but they have not yet eclipsed conventional ELISA methods in popularity. While simultaneous measurement of multiple analytes has, in theory, enormous potential to increase efficiency and reduce costs in a variety of contexts, the obstacles to adopting these new methods have been substantial. Both solid and liquid bead-based multiplex arrays are commercially available from a variety of vendors and are in use by many high-income country laboratories. However, bead-based arrays are complex to operate and maintain, requiring significant user training and a vacuum system or centrifuge to wash the beads. In addition, sheath fluid and other critical associated consumables increase costs and are potentially difficult to supply to developing countries. Further, the clumping of beads blocking the flow sheath is a common problem when used incorrectly. The planar arrays have similar requirements to the Quansys assay which leverage upon equipment also used for conventional ELISAs, such as a plate shaker, washers and pipettors. However, the core equipment is much more expensive and the timely maintenance of complex optical or electrical instruments in austere settings is a concern.

The Quansys Biosciences multiplex technology overcomes some, but not all, of these difficulties. The assay principle is familiar to those practiced in conventional ELISA, and thus the steps involved in executing the assay are familiar to laboratory staff. Quantification of the chemiluminescent signal the kits generate requires relatively simple equipment. Quansys offers an imager (Q-View) but other types of imager may be used, like those used for other common laboratory methods yielding a chemiluminescent end points, like western blots. In contrast to the Luminex, for example, which requires extensive training in instrument operation and routine maintenance, imagers are, at their most basic, a high pixel intensity digital camera inside a light-proof box, and therefore require minimal training, have no moving parts and do not require routine calibration and/or extensive maintenance. This makes this platform simpler to adopt, and is better-suited to developing country laboratories where instruments may be subjected to imperfect climate control and access to basic laboratory consumables, calibration materials and maintenance contracts are limited. The Quansys system also has advantages where electrical power service may be frequently interrupted. Bead-based multiplex assay techniques rely on relatively time-consuming data acquisition processes, whereas the Quansys plate image capture is relatively quick (under 3 minutes for the entire plate), and the chemiluminescent reaction used to quantify the assay signal is stable for several hours — long enough to allow the operator to restart the plate imaging process in the event of a power interruption.

The panel of analytes included in the MMAT represent those commonly used in surveillance efforts, such as the Demographic and Health Surveys (DHS) Program [35], [40], [45], aimed at gauging prevalence of two common MN concerns: IDA and VAD [5]. Because studies of this kind rarely measure just one analyte, bundling the assays into a single, multiplexed kit provides an effective and elegant tool for projects of this kind. However, the DHS and others are increasingly responding to a need to increase the list of biomarkers measured in these large data collection efforts. The Quansys multiplex platform currently can in principle incorporate up to 25 analytes to be measured simultaneously. Our intention is that the MMAT panel described in this work could be expanded to include other assays, including other MN deficiency markers and biomarkers for a number of common infectious diseases.

Adapting this panel for use with dried blood spot specimens (DBS) would also improve the usefulness of the MMAT for large-scale public health surveillance projects. Because ferritin cannot be measured accurately in DBS [46], it would be necessary to include an alternative indicator of iron status, such as hepcidin [47]. The other four markers in the existing panel have been measured successfully with DBS, as have a number of other markers of infectious diseases and non-communicable diseases that could potentially be added to this multiplex panel. Multiplex methods offer a practical solution to the difficulties in assaying multiple analytes using the small sample volumes DBS and other forms of capillary blood collection can yield.

In conclusion, we have developed the MMAT to be a valuable tool for practical and efficient assessment of a panel of analytes related to iron and vitamin A assessment at greatly reduced cost compared to current methods. The simplicity of this multiplex method makes it a better fit than other multiplex platforms for large-scale public health projects, particularly those conducted in low to middle income countries. This assay platform has excellent potential for future expansion for use with DBS and to add more markers relevant to global health concerns.
